# Temporal trends of mercury levels in fish (dab, *Limanda limanda*) and sediment from the German Bight (North Sea) in the period 1995–2020

**DOI:** 10.1007/s10661-022-10655-y

**Published:** 2022-11-05

**Authors:** Ulrike Kammann, Pedro Nogueira, Maike Siegmund, Nicole Schmidt, Stefan Schmolke, Torben Kirchgeorg, Matthias Hasenbein, Klaus Wysujack

**Affiliations:** 1 Thünen Institute of Fisheries Ecology, Herwigstraße 31, 27572 Bremerhaven, Germany; 2 Federal Maritime and Hydrographic Agency, Wüstland 2, 22589 Hamburg, Germany

**Keywords:** Bioaccumulation, Marine monitoring, Flatfish, Age, Time trend

## Abstract

As a toxic and harmful global pollutant, mercury (Hg) enters the marine environment through natural sources, and human activities. It bioaccumulates through the food chain and therefore, Hg is of great importance for environmental monitoring. This study aims to answer the question if Hg contamination in fish and sediment from the German Bight follows temporal trends. Therefore, 496 individual female dab (*Limanda limanda*) were analyzed. The Hg concentrations in the muscle of dab from the German Bight showed significant increase in function of time with an annual percental change of 1.4%, leading to a 41% increase in Hg contamination level within 25 years of monitoring. At the same time, Hg concentrations in sediment—analyzed in 86 samples—significantly decreased in the nearby North Sea environment. This surprising contradiction is shown in the present study and possible causes are discussed. It could be clearly shown that contamination in sediment and biota can follow completely different time courses and therefore, different environmental matrices should be considered in future monitoring studies. Age of the fish turned out to be a biological factor of particular importance for temporal trend analysis.

**Supplementary Information:**

The online version contains supplementary material available at 10.1007/s10661-022-10655-y.

## Introduction

Mercury (Hg) is ubiquitous in the marine environment and at the same time considered one of the most toxic elements or substances on the planet. Hg has outstanding physical properties: It is liquid at room temperature in the elementary form and has a relatively high vapor pressure at the same time. Hg is released from anthropogenic as well as from natural sources (Clarkson & Magos, 2006). In the atmosphere, Hg can stay for about a year—consequently, Hg emissions are distributed globally via atmospheric transport before they reach, e.g., the oceans with the rain (Li et al, 2020). Well known is the direct atmospheric deposition of Hg, which is regarded as a major contamination source for the seas (Driscoll et al., 2013).

Marine sediments are an important sink for Hg—accumulating half of the anthropogenic Hg in the oceans (Covelli et al., 2021; Zhang et al., 2014). In addition, marine sediments have been described as a long-term repository with massive capacities for Hg. Oceans are known to contain more Hg than the atmosphere and surface soils combined (Outridge et al., 2018). Due to their nature, sediments are perfect for the temporal and spatial integration of pollution. Thus, they are perfectly suitable for the comparison of pollution in different areas and ideal for the identification for sources of contaminants.

Hg in water can be converted into methylmercury (MeHg) by chemical processes as well as by microorganisms. MeHg is the most toxic organic Hg species and has the ability to cause environmental and health effects in wildlife (Córdoba-Tovar et al., 2022; Driscoll et al., 2013) as well as to biomagnify along food chains (Lavoie et al., 2010). Since MeHg is a potent neurotoxin, the central nervous system is the most vulnerable organ in mammals (Novo et al., 2021). Understanding the mechanisms of bioaccumulation is crucial to predict food webs at risk for higher rates of bioaccumulation that endanger the upper-trophic predators, including humans (McIntyre & Beauchamp, 2007). Hg exposure can be important for the local consumer including recreational anglers who eat their catch; people who prefer to eat local seafood can also be at risk of elevated Hg exposure (Chan & Receveur, 2000). According to WHO, World Health Organization (2021) in fishing regions of countries such as Brazil, Colombia, China, and Greenland, 17 out of thousand children suffer mental disabilities related to the consumption of Hg-contaminated fish. MeHg is largely responsible for the accumulation of Hg in organisms. Lang et al. (2017) showed that 94% of the total Hg quantified in fish muscle was MeHg. Hg and MeHg can induce a variety of adverse effects in fish at physiologic, histologic, bio-chemical, enzymatic, and genetic levels (Morcillo et al., 2017), partly at environmentally realistic concentrations. Lang et al. (2017) reported a statistical correlation between disease prevalence in dab (*Limanda limanda*) and the Hg environmental concentrations in the North Sea. The authors thus suggested that Hg may affect the health status of fish.

Dab is one of the most suitable fish species for environmental monitoring in the North Sea due to its benthic lifestyle and its geographically widespread distribution. Additionally, dab is considered to be a relatively stationary species. This species has been previously used as a bioindicator in several studies, e.g., on heavy metals (Kammann et al., 2021; Lang et al., 2017) or organic contaminants (Kammann, 2007; Kammann et al., 2017). Furthermore, according to the OSPAR CEMP, dab is the first-choice flatfish species for chemical monitoring (OSPAR, 2018). The European Marine Strategy Framework Directive (MSFD) aimed for establishing a good environmental status of European marine waters by 2020. Under Descriptors 8 and 9, MSFD refers to trace metals including Hg (Law et al., 2010). Monitoring, especially when time trends can be established, is the prerequisite for predictions of contamination rates in different matrices and accumulation of toxic substances in biota as well as in sediment and thus for risk assessment in the environment.

Even if Hg is monitored for a long time in the marine environment, there is a knowledge gap in the evaluation of those longer time series and in trend analyses of North Sea fish. There is also a need for studies linking contamination of fish with sediment contamination in the same area and including supplementary biological factors. Therefore, the present study aims to answer the following questions:

1. Do Hg concentrations in dab and sediment from the German Bight show temporal trends?
2. How does bioaccumulation influence Hg concentrations in dab?
3. Which environmental factors are important for the temporal trend assessment?

## Material and methods

### Sampling

**Fig. 1:**
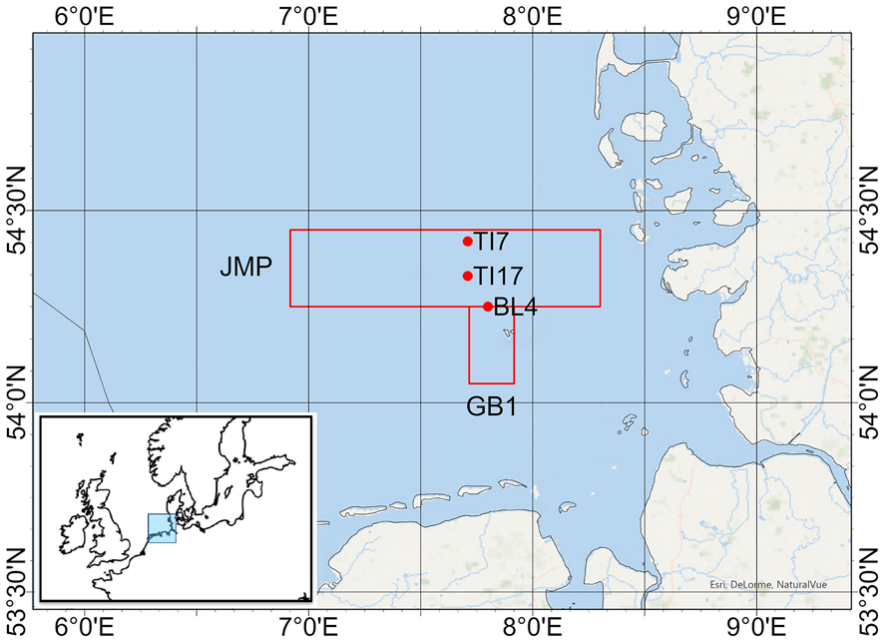
Sampling sites of dab (squares) and sediment (dots) in the German Bight/North Sea (sources of the basemap: Esri, Garmin, USGS, NPS); the small map indicates the location of the big map

Fish were sampled at two and sediment at three stations in the North Sea/German Bight with a spatial overlap (Fig. 1). Dab (*Limanda limanda*) were collected during 20 cruises of RV Walther Herwig III, RV Solea, and RV Uthörn between 1995 and 2020 by bottom trawling (GOV, 30–60 min towing time at 3–4 knots). Sampling took place in August or September, except 1996 and 2010 where the sampling month was October. Geographical coordinates for fish sampling were as follows: JMP 54°15.00ʹN–54°27.00ʹN, 6°55.00ʹE–8°18.00ʹE; GB1 54°03.00ʹN–54°15.00ʹN, 7°43.00ʹE–7°55.00ʹE. Detailed cruise information is provided in Table 1. A total 496 live female dab with 16 to 26 cm total length were sorted from the catches and kept alive in tanks with running seawater of ambient water temperature prior to dissection. Fish were weighted, the total length was measured, sex was visually determined, and animals were anesthetized by a blow on the head, followed by decapitation. The skin was partly removed, and a portion of muscle filet of individual fish was collected with a ceramic knife and stored frozen in plastic tubes precleared with nitric acid and stored at − 20 °C until further processing. Biometric data were used to determine Fulton ‘s condition factor (CF = weight [g] * 100/length [cm]^3^) as an indicator of the general fish health status. Otoliths were removed for subsequent age determination according to Maier (1906) and Bohl (1957). All biometric data characterizing the fish are presented in Table 1. Sediment samples were taken during monitoring cruises on BSH research vessels using a box corer (stainless steel box, 17 × 10 × 20 cm, width × length × height). The upper 2–3 cm of the undisturbed sample surface was collected for analysis. Only plastic or titanium tools were applied to avoid contamination. Immediately after sampling, the sediment samples were frozen at − 18 °C in polypropylene boxes until further processing in the laboratory onshore.

**Table 1:** Cruise information and biometric and mercury data of female dab caught at sites JMP (54°15.00ʹN–54°27.00ʹN, 6°55.00ʹE–8°18.00ʹE) and GB1 (54°03.00ʹN–54°15.00ʹN, 7°43.00ʹE–7°55.00ʹE). Total length [cm], weight [g], age [y], condition factor (CF), and mercury (Hg) [µg/kg] in fish muscle related to wet weight (ww) are expressed as mean values with minima and maxima in brackets. Mercury (Hg) [µg/kg ww] in fish muscle is given also as median values. The cruise codes indicate the three ships taking the samples: “SOL”: RV Solea, “WH”: RV Walther Herwig III and RV Uthörn, n.d.: not determined

Year	Site	Date	Cruise	*N*	Length [cm]	Weight [g]	Age [y]	CF	Mean Hg [µg/kg ww]	Median Hg [µg/kg ww]
1995	JMP	01.09.1995	SOL376	25	20.9 (18.5–29.5)	96.5 (64.1–189.3)	2.9 (2–4)	1.02 (0.90–1.21)	60.00 (33.1–138.7)	55.20
1996	JMP	16.10.1996	Uthörn 134	25	22.3 (19.5–24.2)	114.9 (84.1–146.0)	3.9 (3–5)	1.03 (0.91–1.17)	92.04 (59.0–203.0)	81.90
1997	JMP	25.09.1997	WH188	20	21.5 (18.8–26.0)	103.4 (64.8–176.4)	3.3 (2–6)	1.02 (0.90–1.22)	73.69 (35.1–139.5)	62.90
1998	JMP	23.09.1998	WH198	25	24.0 (21.1–25.2)	132.6 (97.6–165.3)	3.4 (3–4)	0.97 (0.80–1.74)	77.84 (38.1–132.5)	72.50
2003	JMP	30.08.2003	WH255	15	n.d	n.d	n.d	n.d	77.04 (34.22–145.93)	62.46
2005	JMP	26.08.2005	WH278	15	21.9 (20.0–23.4)	105.0 (80.0–130.2)	2.7 (2–4)	1.00 (0.87–1.15)	93.07 (54.31–135.02)	90.70
2006	JMP	01.08.2006	WH291	15	23.1 (20.2–24.9)	123.7 (88.9–163.5)	3.2 (2–4)	1.00 (0.89–1.09)	130.86 (52.26–225.37)	125.78
2007	JMP	01.09.2007	WH303	16	n.d	n.d	n.d	n.d	134.89 (22.29–344.06)	125.05
2008	JMP	16.09.2008	WH315	15	21.7 (19.5–25.2)	100.9 (71.5–153.5)	2.7 (2–4)	0.96 (0.84–1.06)	99.45 (55.75–181.61)	100.27
2009	JMP	07.09.2009	WH325	12	20.8 (19.0–24.8)	92.8 (61.9–145.3)	2.9 (2–5)	1.00 (0.90–1.12)	109.19 (48.51–302.22)	92.05
2010	JMP	07.10.2010	WH338	14	20.3 (16.6–26.5)	84.4 (51.3–159.0)	n.d	0.98 (0.85–1.12)	69.74 (17.23–118.00)	63.14
2011	JMP	08.09.2011	WH346	15	22.5 (20.2–25.8)	111.1 (83.5–165.3)	3.0 (2–4)	0.97 (0.79–1.07)	107.49 (55.70–218.53)	92.68
2012	JMP	05.09.2012	WH357	15	21.9 (19.7–25.3)	104.7 (73.0–151.5)	3.2 (1–5)	0.99 (0.84–1.18)	103.83 (49.33–184.24)	96.55
2013	JMP	08.09.2013	WH367	15	20.9 (19.9–22.2)	94.5 (74.0–113.4)	2.5 (2–4)	1.03 (0.93–1.10)	111.85 (57.77–187.96)	98.56
2014	JMP	10.09.2014	WH377	26	22.1 (20.0–26.2)	100.9 (69.1–157.1)	3.3 (2–5)	0.92 (0.82–1.05)	152.60 (60.23–298.99)	131.91
2015	JMP	14.09.2015	WH387	15	20.9 (19.0–23.5)	90.3 (73.2–110.6)	n.d	1.00 (0.83–1.29)	114.35 (51.06–222.44)	105.40
2016	JMP	31.08.2016	SOL724	15	22.0 (20.2–25.0)	99.9 (76.5–132.2)	2.5 (2–4)	0.93 (0.82–1.06)	199.23 (116.52–412.91)	188.15
2017	JMP	09.09.2017	WH408	16	19.8 (18.0–23.0)	83.3 (51.0–138.0)	1.8 (1–3)	1.05 (0.80–1.21)	96.62 (54.37–184.22)	83.47
2018	JMP	06.09.2018	WH419	15	18.5 (18.0–19.0)	67.8 (52.0–96.0)	1.3 (1–2)	1.07 (0.89–1.40)	120.75 (52.39–231.95)	99.80
2020	JMP	01.09.2020	WH438	16	20.6 (19.0–23.0)	92.5 (65.0–137.0)	2.1 (2–3)	1.05 (0.89–1.21)	149.15 (60.12–307.18)	146.85
	**JMP all**			**345**	**21.5 (16.6–26.5)**	**101.5 (51.0–189.3)**	**2.9 (1–6)**	**1.00 (0.79–1.74)**	**107.06 (17.23–12.91)**	**95.51**
2007	GB1	06.09.2007	WH303	15	21.2 (19.9–24.6)	92.7 (70.1–123.3)	2.6 (2–5)	0.97 (0.81–1.08)	89.34 (17.10–342.75)	64.08
2008	GB1	17.09.2008	WH315	15	21.4 (19.7–24.8)	97.9 (71.0–154.9)	2.1 (2–3)	0.99 (0.81–1.03)	85.31 (57.36–132.61)	76.48
2009	GB1	06.09.2009	WH325	11	20.5 (18.5–24.2)	91.8 (65.5–156.13)	2.9 (2–4)	1.04 (0.87–1.11)	142.94 (62.85–317.88)	138.26
2012	GB1	04.09.2012	WH357	15	21.0 (19.0–23.5)	93.5 (72.0–119.5)	2.7 (2–4)	1.01 (0.69–1.26)	120.49 (57.17–213.15)	106.78
2013	GB1	07.09.2013	WH367	15	21.3 (18.8–25.0)	99.6 (68.1–151.4)	2.5 (1–4)	1.02 (0.84–1.20)	116.43 (57.35–226.52)	121.37
2015	GB1	15.09.2015	WH387	17	21.5 (19.5–24.0)	97.2 (67.5–125.1)	n.d	0.98 (0.88–1.08)	161.09 (50.14–308.42)	150.89
2017	GB1	10.09.2017	WH408	16	18.9 (18.0–22.0)	70.0 (57.0–111.0)	1.9 (1–4)	1.03 (0.85–1.20)	117.35 (49.37–242.25)	110.64
2018	GB1	07.09.2018	WH419	15	18.6 (18.0–19.0)	67.7 (58.0–85.0)	1.4 (1–2)	1.05 (0.92–1.24)	120.60 (68.48–155.23)	122.76
2019	GB1	30.08.2019	WH429	16	20.3 (18.0–23.0)	80.9 (54.0–120.0)	n.d	0.97 (0.81–1.14)	222.58 (123.23–433.42)	222.17
2020	GB1	02.09.2020	WH438	16	20.8 (19.0–24.0)	91.3 (74.0–129.0)	2.1 (1–3)	1.02 (0.83–1.12)	177.14 (91.64–396.99)	165.20
	**GB1 all**			**151**	**20.5 (18.0**–**25.0)**	**88.1 (54.0**–**156.1)**	**2.2 (1**–**5)**	**1.01 (0.69**–**1.26)**	**136.20 (17.10**–**433.42)**	**125.20**

### Analytical methods

The determination of Hg in fish from 1995 to 2009 followed the protocol described by Harms (1981, 1998). Briefly, wet samples were subjected to a pressure digestion by nitric acid in PTFE tubes heated up to 135 °C for 2 h. This was partly supported by a microwave system, “MLS-Ethos-plus” (MLS Mikrowellen Labor-Systeme GmbH, Leutkirch, Germany). Mercury was determined by cold vapor atomic absorption spectrometry using a flow injection mercury system Model 420 (Perkin Elmer, Rodgau, Germany) with background compensation and Hg lamp. A clean bench was used to avoid contamination during sample processing. The limit of detection (LD) was calculated according to DIN 32,645 (DIN, 1994) and was always below 1.2 µg Hg/kg wet weight (ww). The method used from 2010 to determine Hg in fish is in detail described in Kammann et al. (2021). Briefly, samples were freeze-dried using a lyophilizer (LD 1–2, Christ, Osterode, Germany) and subsequently homogenized using an agate mortar or an ultra Turrax tube drive dispenser (IKA, Staufen, Germany) to obtain a dry and homogenous sample powder. Total Hg was determined by atomic absorption spectrometry using a Direct Mercury Analyzer (DMA-80, MLS, Leutkirch, Germany). A total of 20–30 mg of each sample were weighted into the boat containers of the DMA-80. Direct analysis for total Hg content was performed using a 10-level calibration with standards in 0.5 M nitric acid. The LD and the limit of quantification (LQ) were calculated from a standard curve according to DIN 32,645 (DIN, 1994) with a confidence level of 99%. Considering the sample preparation, a LD of 0.080 µg/kg ww and a LQ of 0.230 µg/kg ww were determined for Hg. No values below these limits were found in any sample under investigation. Preparation steps were carried out under clean benches all the time. Clean lab conditions of ISO class 7 were available since 2018. Nitric acid (69%, ultrapure quality) and certified standard solutions of Hg were purchased from Carl Roth, Karlsruhe, Germany, in 0.5 M nitric acid. Ultrapure water was obtained from a Purelab Flex 3 device (Elga Veolia; High Wycombe, UK).

Analysis of Hg in sediment was performed by the German Federal Maritime and Hydrographic Agency (BSH, Hamburg, Germany) as part of the German marine routine pollution monitoring program. Frozen samples were carefully freeze-dried without heat and using a vacuum (0.1 mbar) in order to directly evaporate ice residuals. Subsequently, the dried sediment samples were sieved wet in an ultrasonic bath over nylon sieves in a multistage process (2000 µm, 60 µm, 20 µm). The obtained fine fractions (< 20 µm) of the sediment samples were freeze-dried again, homogenized by an agate ball mill, and decomposed under pressure in PTFE tubes applying a partial digestion method with nitric acid (65%, Suprapur, Merck). Since 2014, the pressure digestion method was replaced by a microwave digestion system (MARS XPRESS, PFA digestion vessels). Finally, the total mercury content of the samples was determined by atomic absorption spectrometry. A flow injection system (FIAS 400, Perkin Elmer) equipped with an amalgamation unit (conditioned gold platinum net) was applied. The sample preparation was conducted in clean benches and ultrapure water (Merck, Millipore) was used during all sample preparation and measurement operations.

### Analytical quality assurance

The accuracy of the Hg measurement in fish was determined by analysis of Certified Reference Materials (e.g., DORM-4) obtained from the National Research Council (NRC) in Canada which was taken through the same analytical procedure as the samples. All samples were measured in triplicates. External quality assurance was done by participation in laboratory proficiency tests conducted by QUASIMEME (www.wepal.nl) designed for marine environment analytics from 1997 to 2021. Results of 55 QUASIMEME intercalibrations are presented in Table S1 with a mean *z*-score of 0.09 and only 7% of unsatisfying results (*z*-score >  = │2.5│). With this successful quality assurance over a long period of time, accuracy of results is clearly documented even while the analytical protocol has changed.

The applied method for sediment is accredited according to EN DIN ISO 17025 and is regularly externally assessed. The accuracy of the method was verified by the regular measurement of standard reference materials (NRC-CNRC “MESS-3”) during the sample batch processing. Furthermore, the method regularly (every year) participates in interlaboratory proficiency tests of marine samples under the QUASIMEME framework (https://www.wepal.nl). During the period 1996 to 2019, 49 QUASIMEME marine sediment test materials (MS1) were analyzed. The laboratory performance tests were in all cases successful with a mean *z*-score of 0.4 and a maximum *z*-score of 1.1.

### Statistics and calculations

Statistical analyses were carried out using Statistica Version 12.5 (StatSoft Europe, Hamburg Germany). The correlation between the concentration of Hg in the muscle and the age of fish as well as between condition factor and Hg in the muscle was tested using a linear regression. For site differences, univariate ANOVA was used. The trends in Hg concentrations were evaluated through the non-parametric Mann–Kendall rank test (MK) (Gilbert, 1987) for monotonic trends. Medians of individual measurements on a yearly basis were used as input to the MK because medians are more robust to outliers than means. The trend analysis was conducted using the radtest package in R (R Core Team, 2013). The annual percent change (APC) in Hg concentration was calculated from log-transformed individual data using the equation (*e*^*B*^ − 1) × 100, where *B* is the slope of the linear trend line between log Hg concentration and sampling year (Hirsch et al., 1991).

## Results and discussion

Fish from two study sites in the German Bight (Fig. 1) were included in this study. In total, 496 dab were sampled during 20 cruises in a 25-year period and individual muscle samples were analyzed for Hg. All samples exhibited Hg concentrations above LOQ. Mean Hg concentration was 107.06 µg/kg ww in JMP and 136.20 µg/kg in GB1, and individual concentration ranged from 17.10 to 433.42 µg/kg ww. The highest Hg yearly mean concentration was found in 2019 in GB1 with 222.58 µg/kg ww. The lowest yearly mean concentration was reported in 1999 in JMP with 60.00 µg/kg ww. The mean length of fish from JMP was 21.5 cm (20.5 cm at GB1). The mean weight of all fish from JMP was 101.5 g (88.5 g at GB1) and the mean age in fish from JMP was 2.9 years (2.2 years at GB1). The mean value of the condition factor (CF) in fish from JMP and GB1 was 1.0 and ranged between 0.69 and 1.74 in the individual fish throughout the period of investigation. Detailed information is provided in Table 1.

Hg concentrations as well as age of fish from the two sites JMP and GB1 were highly significantly different (*p* < 0.001). However, bioaccumulation (Hg increase with age) was rather similar at both sites when the same time frame (2007 and later) was considered (results not shown). This indicates that the observed Hg differences are mainly explainable by the age. CF did not show difference between the two sites.

Surface sediment samples from the German Bight matching the origin of the dab under investigation (Fig. 1) were included in this study. In total, 86 sediment samples collected from 1995 to 2018 were analyzed for Hg in the finest fraction (< 20 µm). Until 2003, Table 2 provides means from two or more individual samples per site and year. From 2004 onwards, only one sample per site and year was analyzed. Mean Hg concentration over the whole period was 0.292 mg/kg dw. The highest Hg mean concentration per site was found in 1995 at BL4 with 0.433 mg/kg dw. The lowest concentration per site was reported in 2012 at TI7 with 0.157 mg/kg dw. Detailed information is provided in Table 2.

**Table 2:** Mercury data in surface sediment in < 20 µm fraction analyzed by the German Federal Maritime and Hydrographic Agency (Hamburg, Germany). For further information, compare text

Year	Site	Mean Hg [mg/kg dw]	*N*
1995	Ti7	0.303	2
	BL4	0.433	1
	Ti17	0.353	2
1996	Ti7	0.390	2
	BL4	0.410	2
	Ti17	0.362	2
1997	Ti7	0.325	2
	Ti17	0.400	2
1998	Ti7	0.325	2
	BL4	0.392	2
	Ti17	0.369	2
1999	Ti7	0.257	2
	BL4	0.400	2
	Ti17	0.284	2
2000	Ti7	0.272	2
	Ti17	0.338	2
2001	Ti7	0.248	2
	BL4	0.390	2
	Ti17	0.277	2
2002	Ti7	0.242	3
	BL4	0.334	5
	Ti17	0.279	3
2003	Ti7	0.221	2
	BL4	0.284	2
	Ti17	0.269	2
2004	Ti7	0.233	1
	BL4	0.337	2
	Ti17	0.266	1
2005	Ti7	0.245	1
	BL4	0.262	1
2006	Ti7	0.227	1
	BL4	0.248	1
	Ti17	0.250	1
2007	Ti7	0.225	1
	BL4	0.270	1
	Ti17	0.285	1
2008	Ti7	0.214	1
2009	Ti7	0.243	1
	BL4	0.237	1
	Ti17	0.239	1
2010	Ti7	0.171	1
	BL4	0.254	1
	Ti17	0.286	1
2011	Ti7	0.165	1
	BL4	0.225	1
	Ti17	0.293	1
2012	Ti7	0.157	1
2013	Ti17	0.217	1
2014	BL4	0.238	1
	Ti17	0.213	1
2015	BL4	0.212	1
	Ti17	0.183	1
2016	Ti17	0.201	1
2017	Ti7	0.237	1
	Ti17	0.264	1
2018	Ti17	0.284	1
All		0.292	86

**Fig. 2:**
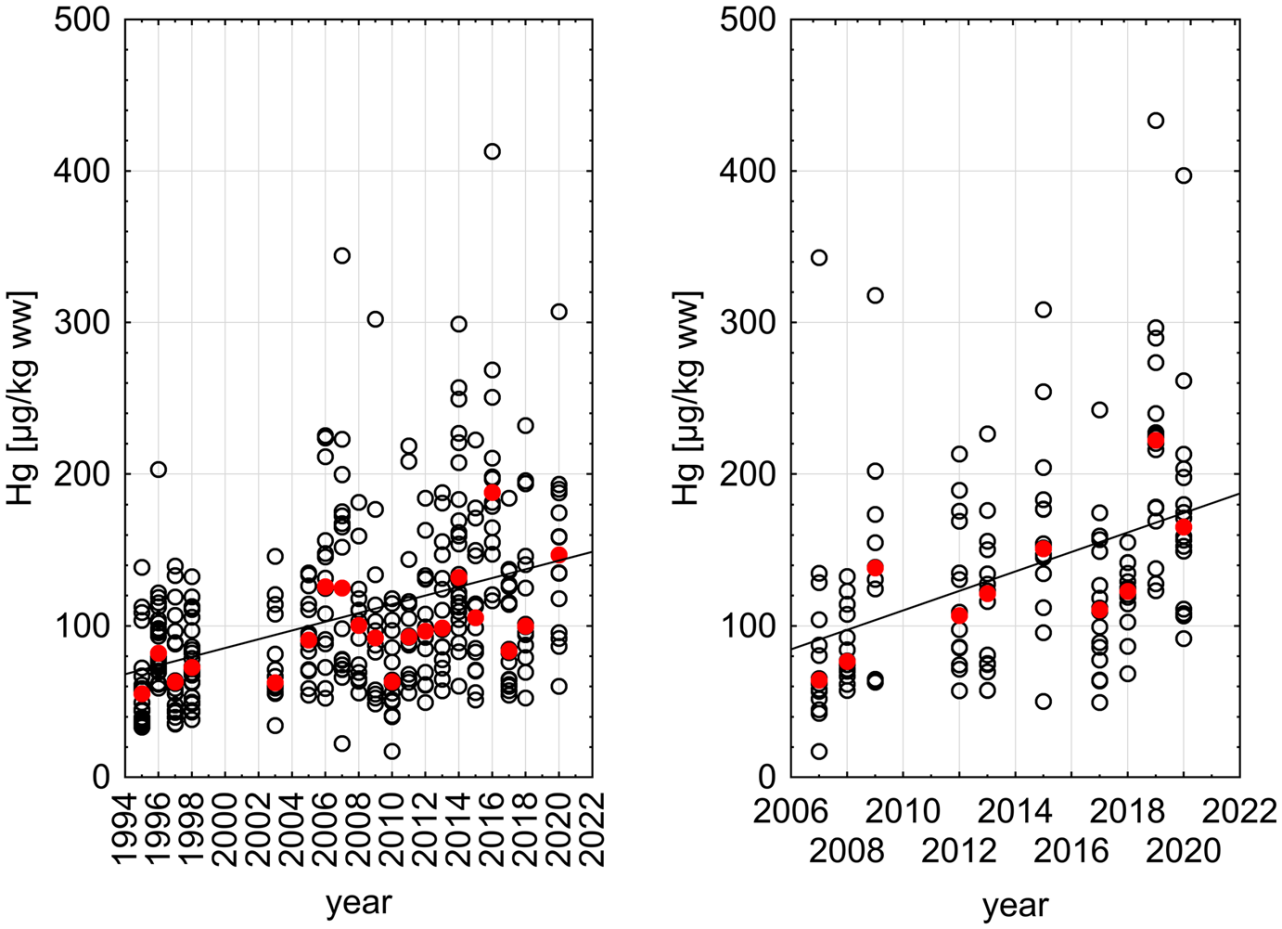
Relation between Hg concentration [µg/kg ww] in dab muscle and sampling year in the German Bight/North Sea. Given are the linear correlations (solid line) for individual values (open circles) as well as medians (red dots) for each year (basis for the Mann–Kendall test). Left: site JMP; right: site GB1. Equations: left: Hg =  − 5686 + 2.886 * year; right: Hg =  − 12,827 + 6.437 * year

**Fig. 3:**
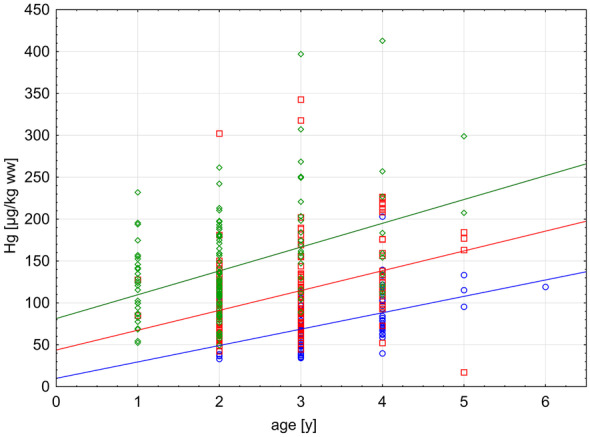
Bioaccumulation of Hg [µg/kg ww] in dab with age. Samples taken from 1995 to 2020 in the German Bight/North Sea (sites JMP and GB1). Given are individual values (open forms) and the linear correlation (solid line) for time intervals: blue (1995–1998), red (2003–2013), and green (2014–2020)

**Fig. 4:**
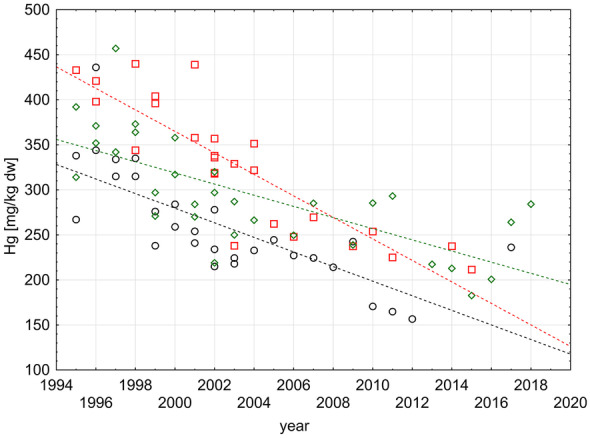
Time series of Hg concentration [mg/kg dw < 20 µm] in sediment and sampling year in the German Bight/North Sea. Given are the linear correlations (dashed line) for individual values (open forms) for the stations TI17 (green), TI7 (black), and BL4 (red). Equations: TI17: Hg = 955 − 0.00002 * year; TI7: Hg = 1099 − 0.00002 * year; BL4: Hg = 1593 − 0.00003 * year. For further information, compare Fig. 1

The Mann–Kendall rank test of Hg median values in fish muscle revealed significant positive trends for both sites (JMP: *n* = 20, *p* = 0.002; GB1: *n* = 10, *p* = 0.010; Table 1). In contrast, statistically significant negative trends were found for Hg in sediments at the three studied sites Ti17, Bl4, and Ti17 (*n* = 21/16/19; *p* < 0.001; Table 2 and Fig. 4). Figure 2 shows the relation between sampling year and the concentrations of Hg in dab at both sites under investigation. The APC for JMP and GB1 together was calculated with 1.4% which results in an increase of 41% within 25 years of monitoring. Both sites have been combined because even if the Hg contamination levels at both sites differ significantly as described above, bioaccumulation (Hg increase with age) was rather similar (Fig. 3).

Bioaccumulation of Hg in dab from contaminated food and/or uptake from the environment induces an increase of Hg concentration with age (Fig. 3). The increase per time (slope of the linear regression in Eqs. (1) to (3)) remains in a comparable range with a minor increase during the different time periods shown in Fig. 3, but the basis level of Hg accumulation (*y*-intercept) increases over time from 9.928 in Eq. (1) to 81.350 in Eq. (3). The differences in the *y*-intercepts reflect the increasing contamination level with time as also shown in Fig. 2. The time periods in Eqs. (1) to (3) were chosen partly for graphical reasons.

1 $$1995-1998:\mathrm{Hg }[\mu \mathrm{g}/\mathrm{kg ww}]=9.928+19.587*\mathrm{age}[\mathrm{y}]$$

2 $$2003-2013:\mathrm{Hg }[\mu \mathrm{g}/\mathrm{kg ww}]=43.721+23.666*\mathrm{age}[\mathrm{y}]$$

3 $$2014-2020:\mathrm{Hg }[\mu \mathrm{g}/\mathrm{kg ww}]=81.350+28.408*\mathrm{age}[\mathrm{y}]$$

The aim of the study was to investigate the development of mercury contamination in fish in the German Bight/North Sea over a period of 25 years and, if present, to prove temporal trends. Biological parameters such as fish age were to be taken into account. Furthermore, fish exposure was to be considered against the background of the total mercury load of marine sediments in the same region.

The range of Hg concentrations in the dab muscle tissue reported in Table 1 is in well accordance with the values found in previous studies (Baeyens et al., 2003; HELCOM, 2018; Kammann et al., 2021; Lang et al., 2017). However, the observed increasing trend of Hg in fish does not meet the expectations: First, mercury concentrations in fish from the German Bight show no trend over the last years in the recent CEMP assessment (OSPAR, 2021a). Second, considering the known decrease in sediment contamination in some regions of the German Bight (OSPAR, 2014, 2021a; Schmolke, 2016) as well as in atmospheric deposition (Wängberg et al., 2007), one would expect fish to mirror this downward development. Surprisingly, the present data show that the opposite is true: Hg concentrations in fish from the two sites in the German Bight increase significantly over the last 25 years (Fig. 2). In the same time period, Hg contamination of sediment in the same area reveals a decreasing temporal trend (Fig. 4).

Hg concentrations in sediment are in accordance with other values reported for nearby areas. For example, Jin et al. (2012) reported values for mercury in surface sediments for the Wadden Sea/Jade Bay ranging between 0.08 and 0.243 mg/kg dw. The Baltic Sea revealed comparable values ranging from 0.01 to 0.341 mg/kg dw (Kwasigroch et al., 2021). Hg concentrations throughout the world differ quite substantially between regions. Compared to Hg concentrations from some other regions in the world (Table 1 in Kwasigroch et al., 2021), values for the North Sea are lower but still significantly exceed the ERL value of 0.15 mg/kg (OSPAR, 2021b).

In several studies, Hg uptake by fish has been investigated before. In a meta-study, Grieb et al. (2020) showed that increasing as well as decreasing trends in Hg concentrations are reported in mainly North American freshwater and in marine fish. Most of the trends reviewed by Grieb et al. (2020) were decreasing—especially regarding marine fish species. One example for decreasing Hg trends is presented by Guns et al. (1992) for North Sea flounder (*Platichthys flesus*) and plaice (*Pleuronectes platessa*). However, the trend described by Guns et al. (1992) refers to samples taken earlier than in the present study and might therefore be not fully comparable. On the other hand, an increasing long-term trend for Hg in marine environment has been observed in cod from the Inner Oslofjord (Norway) from 1984 to 2019 (Green et al., 2019). The observations in the present study are also in agreement with the assessment of OSPAR (2014) in which an increase in Hg concentration in dab from the North Sea since the late 1990s/early 2000s was reported. However, the latest OSPAR CEMP assessment (OSPAR, 2021a) does not show significant Hg trends in North Sea dab using data from 2004 to 2017. Fish samples from the German Environmental Specimen Bank (UBA, 2022) do not show trends for Hg concentrations in the North Sea between 1997 and 2021.

Possible explanations for the observed increasing trend of Hg in dab (Fig. 2) are climate change effects (Camacho et al., 2020), going alongside with intense rainfalls which can lead to increased Hg precipitation. Climate change might also influence phytoplankton growth, leading to increased bioconcentration of Hg by phytoplankton—the lowest trophic level of the food chain (Bełdowska & Kobos, 2016)—and to enhanced production and bioaccumulation of MeHg through the marine food chain (Dijkstra et al., 2013). That climate changes and dietary composition can largely affect the MeHg uptake of Atlantic cod (*Gadus morhua*) has been shown by Schartup et al. (2019): The authors developed a model and showed that an increase of 1 °C in water temperature would result in a 32% increase of MeHg concentration in large cod. The same temperature increase would still lead to higher MeHg concentrations in cod, even if MeHg in seawater declines by 20%. Elevated water temperatures may in addition lead to an increased metabolism in fish and in other aquatic organisms which may be a cause for increased uptake of MeHg. Increasing water temperatures in the north-east Atlantic during the last three decades can be addressed as climate change effect, even if winter bottom water temperatures (increased between 0.1 and 0.3 °C/decade) in the German Bight are so far not statistically significant—however, sea surface temperature rose significantly in the North Sea including the German Bight by 0.1 to 0.5 °C/decade (Dye et al., 2013). Climate change could also cause lowered pH values which lead to elevated release of heavy metals from the sediment (Zhang et al., 2018) as well as to stormwater events which result in higher Hg inputs from, e.g., rivers (Saniewska et al., 2014), and also increased temperature can influence remobilization of contaminants from sediment during flood events (Brinkmann et al., 2014).

We hypothesize that elevated Hg concentrations could be caused by a changed diet of the fish towards more contaminated prey or by enhanced Hg or MeHg bioavailability stored in sediments—possibly related to climate change (Dijkstra et al., 2013; Schartup et al., 2019). Further studies are needed to confirm this hypothesis.

Trend analysis is one important aspect of contaminant monitoring in the marine environment. OSPAR’s Coordinated Environmental Monitoring Programme (CEMP) aims to deliver comparable data from across the OSPAR maritime area including the North Sea. These data are used for trend assessment available online (OSPAR, 2021b). However, trends of bio-accumulative substances in fish can be recognized with certainty when used biota are comparable by size, age, and sex. Especially bioaccumulation with increasing age of the animal is a factor, which can bias, mask, or even fake a trend in the data (Ruus et al., 2017). Increasing Hg concentration in fish muscle with age is a well-known fact (Kammann et al., 2021; Suhareva et al., 2020). It is therefore important to use organisms in a narrow size or age range for trend analysis. During sample collection, information about the age of the individuum is usually not available and substituted by size classes. Size data of organisms should be supported and verified by later age determination, because growth can differ from site to site and in time for various reasons. Examples are shift in nutritional amount or composition as well as differences in temperature influencing metabolic rates in the organism. Another possibility to overcome this problem is normalization of bioaccumulative chemicals to a certain size or age of the fish (Ruus et al., 2017; Suhareva et al., 2020). Results from the present study clearly show that age of the fish is not the cause for the positive trend displayed in Fig. 3, because fish age decreases since 2014 alongside with increasing Hg concentrations (Table 1; Fig. 3). Furthermore, data in Table 1 show that many dab caught at JMP are about 2 to 4 years old (mean age 2.9 years). This age range is linked to an increase in Hg concentration of 41% from 138.2 µg/kg ww (2-year-old dab) to 195.0 µg/kg ww (4-year-old dab) (data calculated using Eq. (3)). Comparing this to the APC of 1.4% change per year leading to 41% increase in Hg contamination level within 25 years of monitoring, it becomes clear that age determination in biota is of high importance for future studies—especially when trends are shallow or data series are short.

## Conclusions

- Hg concentrations in dab muscle at two sites in the German Bight show increasing trends with 1.4% APC resulting in 41% increase of contamination level within 25 years as well as increasing accumulation rates. The positive trend is independent of fish age.
- Hg in sediment decreases since 1995 in the respective area and therefore cannot explain the increase of Hg in dab.
- For trend assessment of Hg in marine environmental monitoring, sediment and biota should be investigated together, because their temporal contamination trends could be contrariwise. Monitoring in both biotic and abiotic environmental compartments is strongly recommended.
- Age is an important biological cofactor when detecting or verifying temporal trends of bioaccumulative substances like Hg in fish.

## Supplementary Information

Below is the link to the electronic supplementary material.

Supplementary file1 (DOCX 68 KB)

## Data Availability

The data used in this research are publicly available from the International Council for the Exploration of the Sea (ICES), accessible via https://www.ices.dk/data/data-portals/Pages/DOME.aspx.
