# Supplementary material for: Temporal trends of mercury levels in fish (dab, Limanda limanda) and sediment from the German Bight (North Sea) in the period 1995–2020
Source: Environ Monit Assess. 2022 Nov 5;195(1):73. doi: 10.1007/s10661-022-10655-y (PMC9637065; doi:10.1007/s10661-022-10655-y)
Supplement: Supplementary file 1 — Supplementary file1 (DOCX 68 KB) [file 10661_2022_10655_MOESM1_ESM.docx]

Table S1: QUASIMEME [24] quality assurance results for Hg trace amounts in 55 biota samples obtained during 1997-2021 from Thünen-Institute of Fisheries Ecology. Z-score >= │2.5│ are classified as not satisfactory.

| year | Excercise | sample ID | z-score |
| --- | --- | --- | --- |
| 1997 | 312 | QTMO32BT | 0.75 |
| 1997 | 312 | QTMO33BT | -0.64 |
| 1997 | 329 | QTMO34BT | -0.70 |
| 1997 | 329 | QTMO35BT | -0.43 |
| 1998 | 346 | QTMO36BT | -1.03 |
| 1998 | 346 | QTMO37BT | -0.76 |
| 1998 | 375 | QTMO38BT | -0.20 |
| 1998 | 375 | QTMO39BT | -1.95 |
| 1998 | 375 | QTMO40BT | -0.24 |
| 1999 | 392 | QTMO41BT | 0.84 |
| 1999 | 392 | QTMO35BT | 0.52 |
| 1999 | 415 | QTMO43BT | 0.38 |
| 1999 | 415 | QTMO44BT | -0.32 |
| 1999 | 456 | QTMO47BT | -0.29 |
| 1999 | 456 | QTMO48BT | -1.64 |
| 2000 | 433 | QTMO45BT | 0.41 |
| 2000 | 433 | QTMO46BT | 0.07 |
| 2001 | 472 | QTMO49BT | 0.55 |
| 2001 | 472 | QTMO50BT | 0.09 |
| 2003 | 586 | QTMO59BT | 0.04 |
| 2003 | 586 | QTMO60BT | 0.28 |
| 2004 | 602 | QTMO61BT | 0.32 |
| 2004 | 602 | QTMO35BT | 2.42 |
| 2004 | 635 | QTMO63BT | 0.08 |
| 2004 | 635 | QTMO64BT | -0.17 |
| 2005 | 651 | QTMO65BT | -0.31 |
| 2005 | 651 | QTMO66BT | -0.06 |
| 2005 | 685 | QTMO67BT | 0.93 |
| 2005 | 685 | QTMO68BT | 0.64 |
| 2006 | 702 | QTMO70BT | 0.40 |
| 2007 | 743 | QTMO73BT | -0.10 |
| 2007 | 743 | QTMO74BT | 0.7 |
| 2008 | 786 | QTM077BT | -0.8 |
| 2008 | 786 | QTM078BT | 2.3 |
| 2008 | 812 | QTM079BT | 2.7 |
| 2008 | 812 | QTM080BT | 0.2 |
| 2009 | 859 | QTM083BT | -0.5 |
| 2010 | 901 | QTM087BT | -0.4 |
| 2010 | 901 | QTM088BT | -0.1 |
| 2011 | 918 | QTM089BT | -0.8 |
| 2011 | 944 | QTM091BT | -0.5 |
| 2011 | 944 | QTM092BT | 2.6 |
| 2013 | 1008 | QTM097BT | -0.2 |
| 2013 | 1008 | QTM098BT | 0.0 |
| 2014 | 1029 | QTM099BT | -0.1 |
| 2014 | 1029 | QTM100BT | 0.0 |
| 2014 | 2014,1 | QTM101BT | -0.0 |
| 2014 | 2014,1 | QTM102BT | -0.2 |
| 2014 | 2014,2 | QTM103BT | 0.0 |
| 2016 | 2016,1 | QTM109BT | -0.82 |
| 2016 | 2016,1 | QTM110BT | 0.05 |
| 2016 | 2016,2 | QTM112BT | 0.0 |
| 2016 | 2016,2 | QTM113BT | -0.4 |
| 2021 | 2021,1 | QTM130BT | 0.8 |
| 2021 | 2021,1 | QTM131BT | 0.6 |
